# Cylinder power progression associated with axial length in young children: a two-year follow-up study

**DOI:** 10.1007/s00417-023-06149-3

**Published:** 2023-07-06

**Authors:** Wei Gong, Jingjing Wang, Bo Zhang, Xian Xu, Haidong Zou, Kun Liu, Xun Xu, Xiangui He, Jiannan Huang

**Affiliations:** 1 Department of Clinical Research, Shanghai Eye Disease Prevention and Treatment Center, Shanghai Eye Hospital, Shanghai Vision Health Center & Shanghai Children Myopia Institute, Shanghai, China; 2 Department of Ophthalmology, Shanghai General Hospital, Shanghai Jiao Tong University School of Medicine, National Clinical Research Center for Eye Diseases, Center of Eye Shanghai Key Laboratory of Ocular Fundus Diseases, Shanghai Engineering Center for Visual Science and Photomedicine, Shanghai, China

**Keywords:** Cylinder power, Axial length, Myopia, Astigmatism, Children and adolescents

## Abstract

**Purpose:**

To describe the association of refraction development and axial length (AL) in young children and provide new insights into the progression of cylinder power.

**Methods:**

Children (2–3 grades) were enrolled from primary schools in Shanghai and followed up for two years. Cycloplegic refraction, AL, and corneal curvature radius were measured. Refraction parameters were compared among groups with different AL, AL1 (AL < 23.5 mm), AL2 (23.5 mm ≤ AL < 24.5 mm), and AL3 (AL ≥ 24.5 mm). Multiple regression analysis was used to explore risk factors of diopter of cylinder (DC) progression.

**Results:**

In total, out of 6891 enrolled children, 5961 participants (7–11 yrs) were included in the final analysis. Over the two-year period, the cylinder power significantly changed, and those with longer AL had more rapid DC progression over the two years (AL1, -0.09 ± 0.35 D; AL2, -0.15 ± 0.39 D; AL3, -0.29 ± 0.44 D) (*P* < 0.001). The change in DC was independently associated with AL at baseline (*P* < 0.001). The proportion of with-the-rule astigmatism increased from 91.3% to 92.1% in AL1 group, from 89.1% to 91.8% in AL2 group and from 87.1% to 92.0% in AL3 group.

**Conclusions:**

Young children with long AL experienced rapid progression of cylinder power. Both the control of myopia progression and attention to the correction of astigmatism are necessary in the health management of children with long AL. The significantly increased AL in participants might contribute to both the extent and direction of astigmatism.

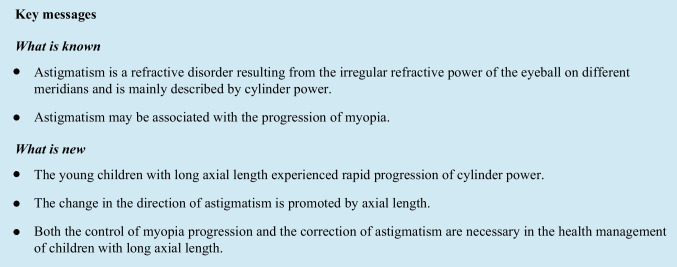

## Introduction

The high prevalence of myopia is a worldwide public health concern. Particularly, in East and Southeast Asia, the prevalence of myopia among high school graduates reaches 80%-90%, with a rate of high myopia of 10%-20% [1–4]. Along with the development of myopia, the elongation of eye axial length (AL) increases the risk of severe fundus complications, which may result in uncorrectable and irreversible vision loss [5–8]. Astigmatism is a refractive disorder resulting from the irregular refractive power of the eyeball on different meridians and is mainly described by cylinder power. Identifying the change patterns of astigmatism associated with AL in children would help understand the influence of AL on astigmatism and provide new insights for the management of myopia. However, to our knowledge, related studies are rare.

Previous studies have reported that the diopter of cylinder (DC) is largely stable during school years. One study including 2242 participants aged 3–18 yrs found that the change in diopter cylinder was -0.02 D/y for a younger cohort (3 to < 11 yrs) and + 0.06 D/y for an older cohort (11 to < 19 yrs) [9]. In another study involving 183 children aged 7–11 yrs, the mean DC changed by + 0.16 D per year [10]. Nevertheless, in clinical practice, it has been observed that severe myopia tends to be accompanied by a progressive absolute value of DC. Our previous study revealed that the average 2-year change in DC in grade 1 to 3 students was + 0.06 D to + 0.02 D, -0.02 D to -0.09 D, and -0.05 D to -0.16 D for the nonmyopia, incipient myopia and persistent myopia groups, respectively [11]. In addition, some studies suggest that astigmatism may be associated with the progression of myopia [12–14].

The effect of AL on the progression rate of DC in children remains unknown. Therefore, our study aimed to describe longitudinal changes in DC in children with AL of different degrees and their influencing factors.

## Methods

### Participants

Children aged 7–11 yrs from grade 2 and 3 grades of 24 schools from eight districts in Shanghai, China, were enrolled in 2016 and followed up for two years. Those with systemic or ocular pathology at baseline, strabismus or amblyopia organic eye were excluded. The study protocol was explained to the participants, and all participants indicated their understanding of the protocol, and oral consent was obtained. Written informed consent forms were obtained from their parents or legal guardians. This research was reviewed by an independent ethical review board and conforms with the principles and applicable guidelines for the protection of human subjects in biomedical research. All participants were treated in accordance with the tenets of the Declaration of Helsinki. Approval from the institutional review board of Shanghai General Hospital, Shanghai Jiao Tong University was obtained (ID: 2015KY149).

### Measurements and calculations

For all participants, a basic examination of height and weight as well as a series of ophthalmic examinations were performed by a well-trained team composed of ophthalmologists, optometrists, coordinators and nurses.

For the safety of cycloplegia, each participant underwent slit lamp examination and intraocular pressure measurement using a noncontact tonometer (NT-510, Nidek, Japan). Those with anterior chamber depth ≥ 2.5 mm and intraocular pressure ≤ 24 mmHg were subjected to cycloplegia as follows: One drop of 0.5% proparacaine (Alcaine, Alcon, USA) was administered to each eye; approximately 15 s later, two drops of 1% cyclopentolate (Cyclogyl, Alcon, USA) were administered, with each drop supplied 5 min apart. At least 30 min after the last drop, pupillary light reflex and pupil diameter were assessed. The absence of light reflection and a pupil diameter larger than 6 mm were considered indicative of complete cycloplegia; otherwise, one more drop of cyclopentolate was administered. During the process, the participants were required to keep their eyes closed to ensure cycloplegia completion.

The refractive status (diopter of sphere (DS); DC; axis, θ; corneal curvature radius, the average value of both meridians) was measured with an autorefractor (KR-8900, Topcon, Japan) after inducing cycloplegia. All instruments were calibrated before examination. Three measurements for each eye were obtained, and the procedure was repeated if the difference between any two records of DC or DS was larger than 0.50 diopter (D).

The AL was measured by an IOL Master instrument (Carl Zeiss Meditec, Germany) after calibration. Each eye was measured three times, and if the difference between any two measurements was greater than 0.05 mm, the measurement was repeated until the difference between 3 consecutive measurements was below this value.

Body mass index (BMI) was calculated with the following formula: BMI = weight (kg)/(height (m))^2^. The spherical equivalent (SE) was calculated as DS + 0.5 * DC. DC and axis (θ) were further converted into Jackson cross-cylinder values (J_0_ = (-DC/2) cos2θ, J_45_ (J_45_ = (-DC/2) sin2θ). The astigmatism was typed according to the cylinder axis. The cylinder axis of 0 ~ 30° and 150 ~ 180° was defined as with-the-rule (WTR), 60 ~ 120° was defined as against-the-rule (ATR), and the other was defined as oblique.

### Statistical analysis

All data were compiled with a self-designed comprehensive database system (Gaussinfomad, Beijing, China) with in-built logic to import data from the autorefractor and IOL-Master. SPSS (version 22.0; IBM Co., Armonk, NY, USA) was used for data analysis. Only data for the right eye were included in the analysis. The data distribution was examined using the Kolmogorov‒Smirnov test. Parameters are presented as the means ± standard deviations for continuous variables and rates (proportions) for categorical data. Comparisons among groups were performed using t tests, analysis of variance, nonparametric tests (non-normally distributed data) or chi-square tests. The t test was used for the comparison of the parameters between baseline and after follow-up. The chi-square test was used for the comparison of proportions. The changes in DC with AL were analyzed by linear regression analysis. Correlations between general characteristics at baseline and one-year changes in refraction parameters were analyzed with Spearman correlation coefficient analysis. Multiple regression analysis was carried out to explore factors associated with longitudinal changes in DC. Statistical significance was set at *P* < 0.05 (two-tailed) (*P* < 0.017 for pairwise comparison). The participants were grouped according to AL as follows: AL1 (AL < 23.5 mm), AL2 (23.5 mm ≤ AL < 24.5 mm), and AL3 (AL ≥ 24.5 mm).

## Results

### General characteristics

Finally, out of 6891 enrolled children, 5977 were followed up for two years, and after excluding those with incomplete data, 5961 participants (7–11 yrs) were included in the final analysis. There was no significant difference in age, gender proportion, SE or AL between the ones included and excluded. Table 1 shows the distributions of the general characteristics and refractive parameters of the AL groups at baseline. There were significantly higher AL, CR, and AL/CR and lower J_0_, J_45_, DS, and SE in those with longer AL (all *P* < 0.001). The AL3 group had the lowest DC (*P* < 0.001).

**Table 1:** General characteristics and refractive parameters by AL groups

Parameters	Total, *n* = 5961	AL1, *n* = 4041	AL2, *n* = 1562	AL3, *n* = 358	*P*-value^b^	Pairwise Comparison^a^
Age (range) (yrs)	8.24 ± 0.61	8.17 ± 0.59	8.36 ± 0.60	8.49 ± 0.53	** < 0.001**	1 < 2 < 3
Gender-boys, n (%)	3147(52.8)	1780(44.0)	1083(69.3)	284(79.3)	** < 0.001**	1 < 2 < 3
Height (cm)	131.19 ± 6.52	130.11 ± 6.32	133.17 ± 6.40	134.84 ± 5.85	** < 0.001**	1 < 2 < 3
Weight (kg)	30.17 ± 8.94	29.19 ± 7.43	31.88 ± 10.10	33.86 ± 15.02	** < 0.001**	1 < 2 < 3
BMI (kg/m^2^)	17.35 ± 4.24	17.09 ± 3.48	17.78 ± 4.78	18.47 ± 7.70	** < 0.001**	1 < 2 < 3
DC (D)	-0.51 ± 0.60	-0.52 ± 0.61	-0.46 ± 0.54	-0.60 ± 0.66	** < 0.001**	3 < 1 < 2
J_0_ (D)	0.21 ± 0.30	0.22 ± 0.31	0.19 ± 0.28	0.24 ± 0.34	**0.001**	1/3 > 2
J_45_ (D)	-0.01 ± 0.13	-0.01 ± 0.13	-0.01 ± 0.12	-0.02 ± 0.15	**0.021**	1 > 3
DS (D)	0.75 ± 1.33	1.17 ± 1.06	0.20 ± 1.14	-1.56 ± 1.62	** < 0.001**	1 > 2 > 3
SE (D)	0.50 ± 1.32	0.91 ± 1.01	-0.03 ± 1.17	-1.86 ± 1.75	** < 0.001**	1 > 2 > 3
AL (mm)	23.16 ± 0.83	22.72 ± 0.53	23.90 ± 0.28	24.97 ± 0.42	** < 0.001**	1 < 2 < 3
CR (mm)	7.85 ± 0.70	7.77 ± 0.82	8.01 ± 0.23	8.08 ± 0.26	** < 0.001**	1 < 2 < 3
AL/CR	2.95 ± 0.15	2.93 ± 0.14	2.98 ± 0.14	3.09 ± 0.20	** < 0.001**	1 < 2 < 3
Astigmatism direction					**0.015**	
WTR, n (%)	5286(90.5)	3684(91.3)	1391(89.2)	311(87.1)		
ATR, n (%)	297(5.0)	191(4.7)	83(5.3)	23(6.4)		
Oblique, n (%)	267(4.5)	159(3.9)	85(5.5)	23(6.4)		

AL1: AL < 23.5 mm; AL2: 23.5 mm ≤ AL < 24.5 mm; AL3: AL ≥ 24.5 mm

^a^Pairwise comparison of the parameters at baseline between each two AL groups, the significant difference (*P* < 0.017) is shown

^b^*P*-value is shown in bold in the comparison with statistical significance

*BMI* Body mass index, *DC* Diopter of cylinder, *DS* Diopter of sphere, *SE* Spherical equivalent, *AL* Axial length, *CR* Corneal curvature radius, *WTR* With-the-rule, *ATR* Against-the-rule

### Change of astigmatism in different AL groups in the two-year follow-up

**Fig. 1:**
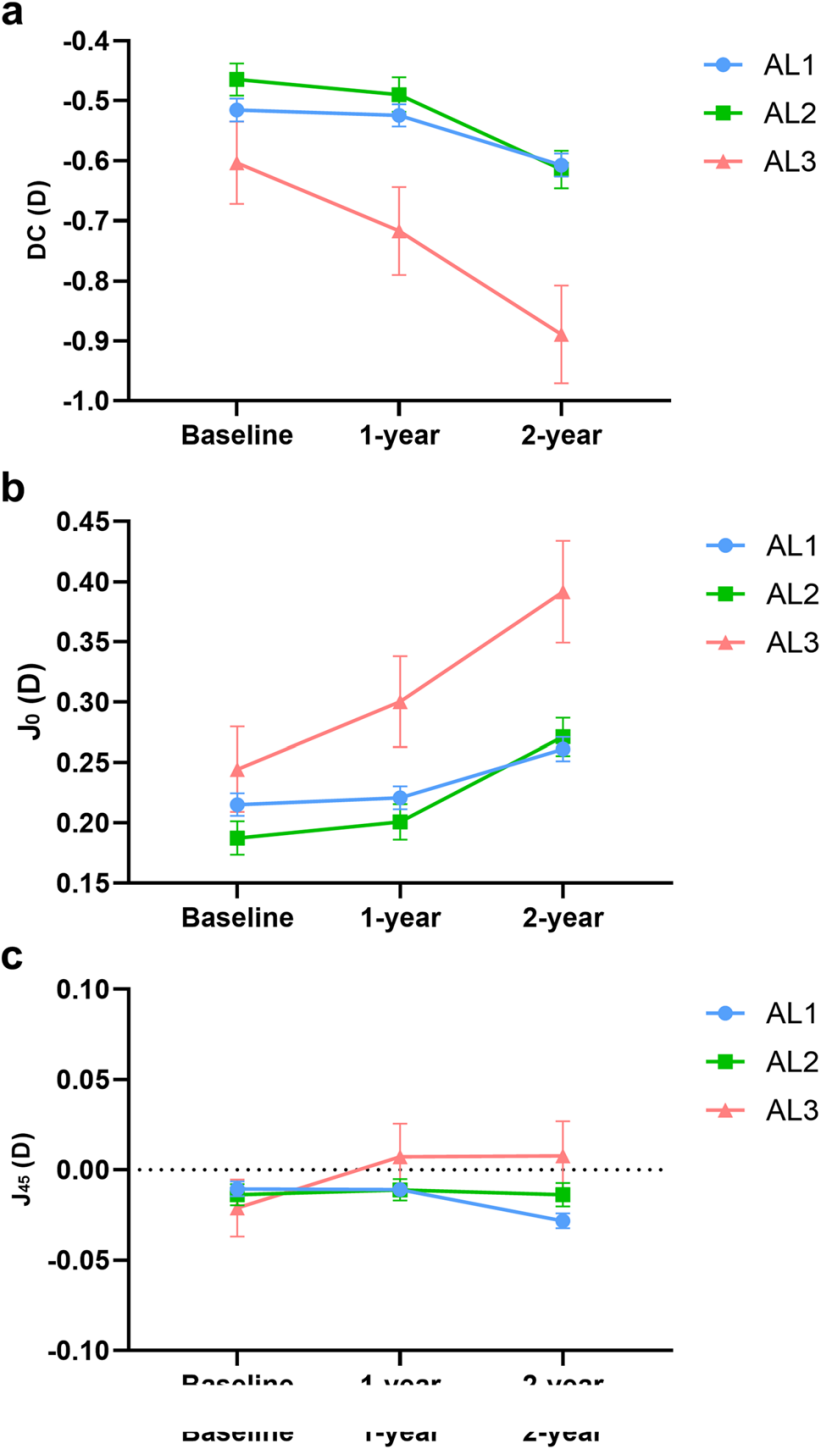
Astigmatism at baseline and at the two-year follow-up. The value and trendline of DC (**a**), J_0_ (**b**) and J_45_ (**c**) at baseline and at the two-year follow-up are shown. Abbreviations and acronyms: DC = diopter of cylinder; D = diopter

Over the two-year period, the cylinder power significantly changed (*P* < 0.001) (Table 2). The difference in the change in DC among the AL groups was significant (*P* < 0.001), and those with longer ALs had more rapid DC progression (AL1 -0.09 ± 0.35 D, AL2 -0.15 ± 0.39 D, AL3 -0.29 ± 0.44 D). As Fig. 1 shows, AL1 had the slowest DC progression (*P* < 0.001). Conversely, AL3 had the fastest DC progression. Similarly, AL3 had the most remarkable change in J_0_ (*P* < 0.001).

**Table 2:** Two-year change of the refractive parameters

Parameters	Total, *n* = 5961	AL1, *n* = 4041	AL2, *n* = 1562	AL3, *n* = 358	*P*-value^b^	Pairwise Comparison^a^
ΔDC	-0.12 ± 0.37	-0.09 ± 0.35	-0.15 ± 0.39	-0.29 ± 0.44	** < 0.001**	1 < 2 < 3
ΔJ_0_	0.06 ± 0.2	0.05 ± 0.19	0.08 ± 0.2	0.15 ± 0.24	** < 0.001**	1 < 2 < 3
ΔJ_45_	-0.01 ± 0.12	-0.02 ± 0.12	0.00 ± 0.12	0.03 ± 0.13	** < 0.001**	1 < 2 < 3
ΔDS	-1.03 ± 0.83	-0.95 ± 0.8	-1.16 ± 0.84	-1.34 ± 0.94	** < 0.001**	1 < 2 < 3
ΔSE	-1.09 ± 0.86	-1.00 ± 0.82	-1.24 ± 0.87	-1.48 ± 0.98	** < 0.001**	1 < 2 < 3
ΔAL	0.66 ± 0.32	0.61 ± 0.31	0.74 ± 0.33	0.85 ± 0.30	** < 0.001**	1 < 2 < 3

AL1: AL < 23.5 mm; AL2: 23.5 mm ≤ AL < 24.5 mm; AL3: AL ≥ 24.5 mm

^a^Comparison of the absolute value of the change of parameters between each two AL groups, the significant difference (*P* < 0.017) is shown

^b^*P*-value is shown in bold in the comparison with statistical significance

*DC* Diopter of cylinder, *DS* Diopter of sphere, *SE* Spherical equivalent, *AL* Axial length

### Changes in AL under different DC progression

**Fig. 2:**
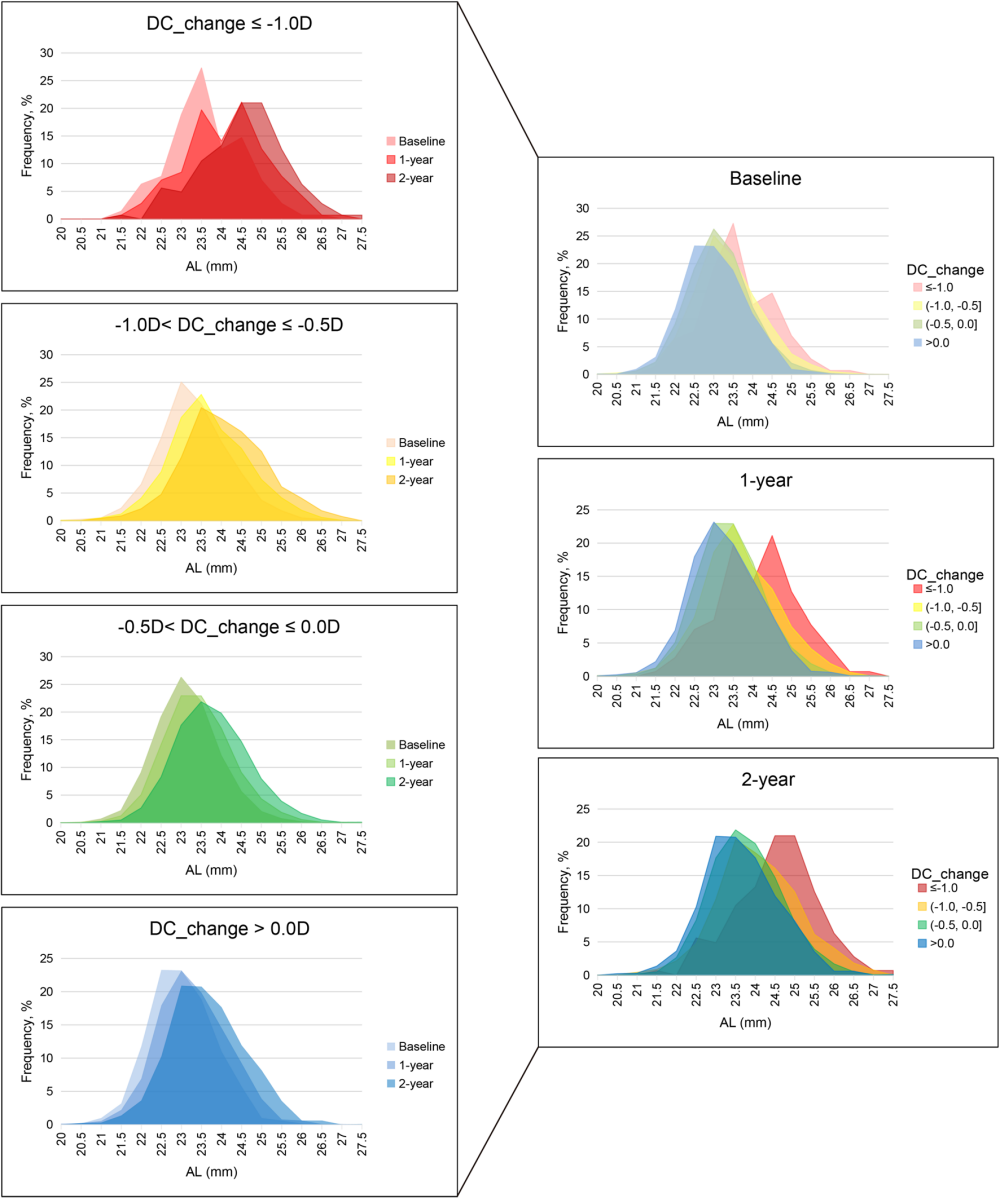
AL at baseline and at the two-year follow-up in the DCchange groups. The frequency of AL at baseline and the follow-up two years in change of DC groups, and the change pattern was exhibited. The participants with more remarkable changes in DC had longer AL (*P* < 0.001). Abbreviations and acronyms: DC = diopter of cylinder; AL = axial length

As Fig. 2 shows, the AL consistently increased with follow-up. For those with more severe astigmatism progression, the AL was longer (all *P* < 0.001). For the children with a change of DC ≤ -1.0 D per year, AL increased from 23.61 ± 0.90 mm at baseline to 24.54 ± 1.04 mm in the third year. For the children with a change of DC > 0 D per year, AL increased from 23.02 ± 0.82 mm at baseline to 23.63 ± 0.94 mm in the third year.

### The change in refraction parameters in the age and AL groups

**Fig. 3:**
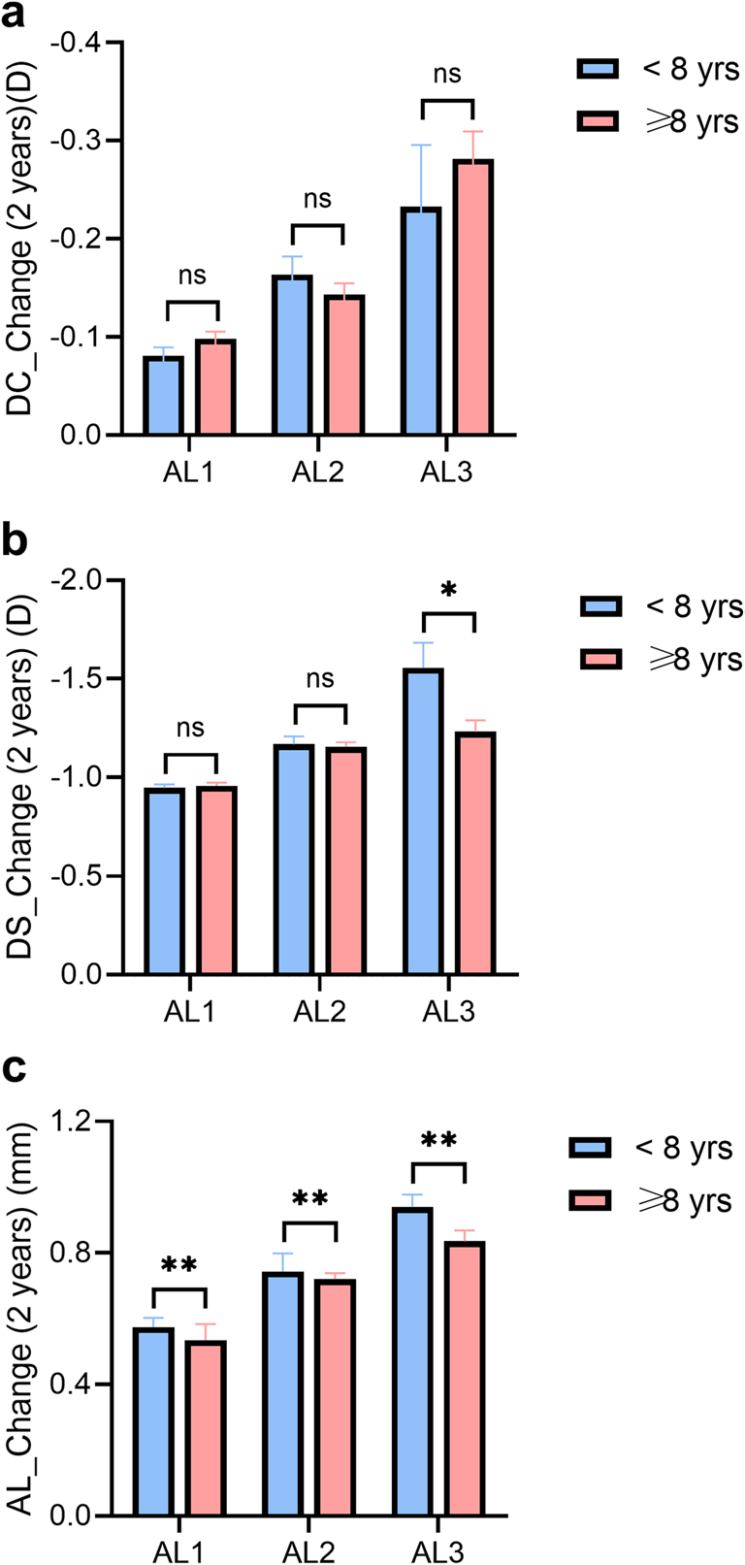
Change in refraction parameters in age groups. The values of change of DC (**a**), DS (**b**) and AL (**c**) were shown. The data were divided according to age (8 years old). The values of the AL groups were compared (ns, P ≥ 0.05; * *P* < 0.05; ** *P* < 0.01). Abbreviations and acronyms: DC = diopter of cylinder; DS = diopter of shpere; D = diopter; AL = axial length

The children were divided into two groups according to age at baseline (2271 participants with age < 8 yrs and 3690 participants with age ≥ 8 yrs) (Fig. 3). There was no significant difference in the change DC in children ≥ 8 years and < 8 years for the AL1-3 groups (all P > 0.05). The change in DS in children ≥ 8 yrs was significantly lower than that in children < 8 yrs for the AL3 groups (*P* < 0.05). The change in AL in children ≥ 8 yrs was significantly lower than that in children < 8 yrs for the AL1-3 groups (all *P* < 0.01).

### Change in direction of astigmatism with follow-up

**Fig. 4:**
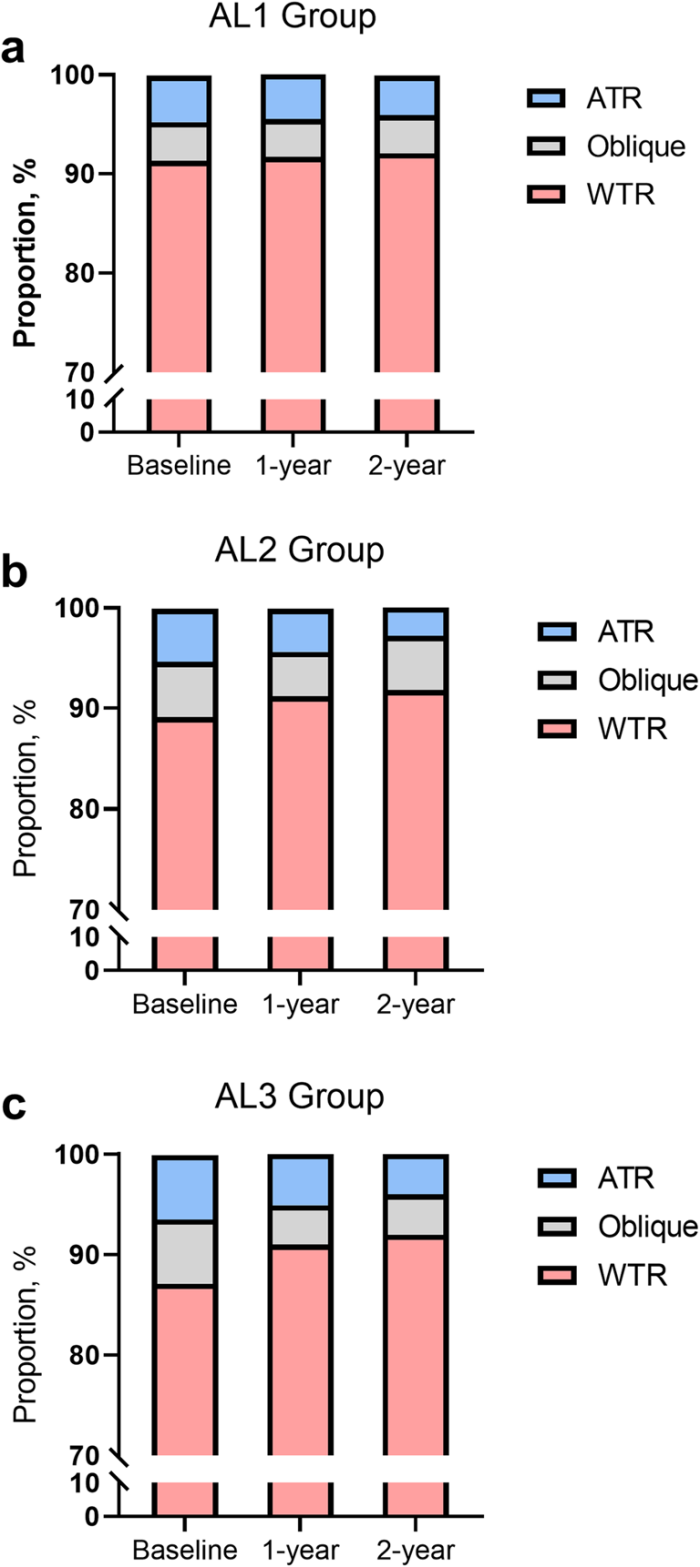
Proportion of astigmatism direction at baseline and at the two-year follow-up. The proportion of astigmatism direction (oblique, ATR, WTR) in AL groups 1 ~ 3 (**a** ~ **c**) at baseline and at the two-year follow-up is shown. Abbreviations and acronyms: WTR = with-the-rule; ATR = against-the-rule

In the two-year follow-up, the proportion of different directions of astigmatism significantly changed (Fig. 4) (*P* < 0.01). The proportion of WTR astigmatism changed from 90.5% at baseline to 92.0% in the third year. The proportion of ATR astigmatism changed from 5.0% at baseline to 3.7% in the third year. The proportion of oblique astigmatism changed from 4.5% at baseline to 4.3% in the third year. The proportion of WTR changed from 87.1% to 92.0% in the AL3 group, from 89.1% to 91.8% in the AL2 group, and from 91.3% to 92.1% in the AL1 group.

### Multiple regression analysis

The multiple regression analysis for the change of DC was conducted (Table 3). After dropping variables with collinearity, greater progression of DC was independently associated with longer AL at baseline (Beta = -0.066; 95%CI, -0.078 ~ -0.054) and milder DC at baseline (Beta = -0.109; 95%CI, -0.125 ~ -0.094) (both *P* < 0.001), but not with gender, age or BMI (all *P* > 0.05).

**Table 3:** Multiple regression analysis for change of DC

	Beta	Standard error	Standard Beta	*P-*value^a^	95%CI	VIF
Constant	1.373	0.142		** < 0.001**	1.094 ~ 1.652	
Gender, boys	0.018	0.010	0.024	0.069	-0.001 ~ 0.038	1.132
Age, yrs	0.001	0.008	0.001	0.916	-0.015 ~ 0.017	1.050
BMI, kg/m^2^	-0.002	0.001	-0.018	0.163	-0.004 ~ 0.001	1.036
AL, mm	-0.066	0.006	-0.149	** < 0.001**	-0.078 ~ -0.054	1.165
DC, D	-0.109	0.008	-0.175	** < 0.001**	-0.125 ~ -0.094	1.009

*BMI* Body mass index, *AL* Axial length, *DC* Diopter of cylinder

^a^*P*-value is shown in bold in the comparison with statistical significance

## Discussion

This study specifically described the association of change patterns of cylinder power and AL in young children. The children in grades II and III had a significant decrease in DC over the two-year follow-up. Those with a long AL at baseline had more rapid astigmatism progression, which accelerated with age. The change in DC per year was independently associated with AL.

The results of our study revealed a meaningful change in the cylinder power of young children with long ALs. On the one hand, the change in DC during the two-year follow-up was significant. On the other hand, the progression of DC in the AL3 group was more remarkable than that in the other groups. Our findings are inconsistent with previous studies indicating that DC remains almost unchanged in school years [9, 10]. A potential explanation for this difference is that the analyses in previous studies were not based on the classification of myopia degree, such as AL [15–17]. According to our findings, more attention should be given to updating the corrective prescription of cylinder power for children with long AL.

Consistent with previous studies [18–20]. our results showed that the shifts of AL and DS slowed in the older participants, but DC remained highly progressive. In addition, the change in DC was not significantly associated with age. These findings suggest that in individuals with long ALs, astigmatism might be stably progressive and difficult to control, which suggests a side effect of AL elongation on refraction development.

In our study, the progression of cylinder power was independently associated with AL. Previous studies have revealed that pressure on the cornea from the eyelids possibly promotes astigmatism in the horizontal meridian [21–28]. Along with the findings in a clinical study [27], we speculated that elongation of the axis length might cause the cornea to protrude more from the orbit and that the influence of the eyelid on the cornea might be strengthened, increasing cylinder power. Accordingly, in our study, the AL3 group had significantly faster progression in the proportion of WTR. In addition, the significant difference in DC among the three groups was mainly due to the difference in J_0,_ supporting our hypothesis. Thus, the significantly increased AL in high myopia might contribute to both the extent and direction of astigmatism. Furthermore, previous studies showed that severe astigmatism could possibly disturb refraction development and promote myopia progression [12, 29]. These findings indicate that AL elongation and astigmatism are intrinsically correlated and should be controlled simultaneously.

There are several limitations to our study. First, the population was relatively limited, from grade II to III of primary school in Shanghai. Our intended future research will cover subjects from a wider age range and more locations. Second, the follow-up rates were only 86.5% in the current study cohorts. However, there was no significant difference in age or sex between the children followed up and those lost to follow-up. Last, the two-year follow-up period of the study was short, which might have resulted in an underestimation of the differences in refraction progression among the groups. A longer observation period is expected to reveal more characteristics in the patterns of cylinder power progression.

Our study specifically describes astigmatism development in children associated with AL and provides new insights into the progression of DC. The young children with long AL experienced rapid progression of cylinder power. In addition, a change in the direction of astigmatism promoted by AL was observed. The findings suggest that both the control of myopia progression and attention to the correction of astigmatism are necessary in the health management of children with long AL.

## Data Availability

The datasets generated during and/or analyzed during the current study are available from the corresponding author on reasonable request.
